# An Inquiry into the Homœopathic Practice of Medicine

**Published:** 1846-04

**Authors:** 


I. An Inquiry into the Homœopathic Practice of Medicine. By *W. Henderson*, M. D., 8vo. pp. 238. 1845.

II. Homœopathy, its Principle, Theory, and Practice. By *M. B. Sampson*. 8vo. pp. 218. 1846.

III. Confessions of an Homœopathist. Small 8vo. pp. 399. 1846.

It is truly lamentable to meet with a publication like the first of these. Within the last 18 months or so, Dr. Henderson was professor of pathology in the University of Edinburgh, and one of the physicians of the Royal Infirmary in that city. He is uow a homœopathic doctor, practising all the petty manœuvres of the Hahnemannian School. As a matter of course, he strives to conciliate the favour and deprecate the censure of his (sometime) brethren, by appealing to *facts*, to successful cases, and marvellous cures effected by the "new method." Drs. Culverwell and Courtenay, and Messrs. Morrison, Holloway, Curtis, &c., could beat him hollow at this game. Many of the cases, which he adduces in the way of illustration, are utterly worthless and contemptible; we should think that some of his (now) *confreres* will themselves be ashamed of them. We can hardly suspect Dr. Henderson of wilfully imposing upon others; that would be dishonest; but that he is wofully deluded is a *fact*, which none but a homœopathist can deny. We much fear that he thinks so himself now. He treats Pneumonia with phosphorus, Hydrocephalus with stramonium and belladonna, and Gastritis with arsenic!

The work of Mr. Sampson is a much more creditable performance; it is alto-

---

gether very well written, and not unfairly reasoned, considering that the author is only an*amateur-medecin*, one who has never been engaged in actual practice. Being an active *lay* member of the "English Homœopathic Association" —which ranks lords and learned doctors, clerks and wealthy merchants, on its committee—he was requested by that body to write a Treatise upon Homœopathy: the present work is the response to this invitation. It contains a good deal that may, somewhat profitably, claim the attention of the medical reader. The main argument of the writer against the "ordinary praetice" of medicine, is based upon the fact of the inconsistency, and, too often, the utter contrariety of treatment recommended by different medical authors for the cure of the same disease. He quotes a variety of extracts from the writings of Craigie, Elliotson, and others in proof of this. Alas! there is too much truth in the charge. But, while we are candid enough to admit the partial force of Mr. S.'s argument upon this point, we must tell him that we cannot perceive an atom of sound reasoning, or of satisfactory evidence, in favour of his favourite system of Homœopathy. As for the fact of "its widest reception being found amongst the shrewdest, the most practical, and, on other than national subjects, the least prejudiced people upon earth—the inhabitants of the United States," we willingly give him the full benefit of its importance. The ingenuity of the analogical illustrations in the subjoined extract will amuse, if not convince, the reader.

"That which renders the ridicule bestowed on the infinitesimal doses, by so-called scientific persons, still more surprising, is, that even the obvious considerations which have just been presented are not needed to give rise to a supposition of their efficacy, since setting aside any process of abstract reasoning, there is enough in the ordinary phenomena of nature to enable the mere observer of daily occurrences to arrive at a conclusion regarding the 'possibility' of these doses proving effective. We all know that a moderate sized pebble may be applied to the surface of the eye without producing any unpleasant effect; while, if the same pebble were reduced to a powder, and one atom of that powder were applied to the same part, the most unendurable symptoms would immediately arise. We know too that persons may often be exposed to an atmosphere abounding with coarse dust, and suffer no injury to their health; while there are some trades in which the workmen are exposed to dust of a similar but much finer kind, which almost invariably induce consumption. Particles of steel, again, are known to produce equally injurious effects; and these effects seem to become more serious in proportion to the power which the particles, by reason of their fineness, may possess of insinuating themselves into the organization." Moreover, "if it can be supposed that diseases such as cancer, &c,, may be the production of an insect not yet detected by the mycroscope, and that this insect may have other beings preying upon it, and these again others, the belief that none but gross and ponderable influences can affect the system must soon become weakened, and the idea that medicines require to be 'finely touched' in order to lead to such 'fine issues' as the destruction of these agencies, must come to appear a natural, instead of a preposterous one."

The "Confessions, &c." is a wretched performance, discreditable alike to the heart and head of the author. Medicine stands in no need of the aid of a bungling novel-writer, who seeks to calumniate those whom she may consider to be mistaken or deceived.

